# First known observations of brooding, development, and hatching of fertilized eggs for the North Pacific bigeye octopus, *Octopus californicus*

**DOI:** 10.1002/ece3.9481

**Published:** 2022-11-03

**Authors:** Adi Khen, Lillian R. McCormick, Christine A. Steinke, Greg W. Rouse, Phil J. Zerofski

**Affiliations:** ^1^ Scripps Institution of Oceanography University of California San Diego La Jolla California USA

**Keywords:** cephalopod embryonic development, deep‐water species, egg‐brooding, North Pacific bigeye octopus, *Octopus californicus*

## Abstract

The North Pacific bigeye octopus, *Octopus californicus* (Berry, 1911) is a cold‐water, deep‐sea octopod. Little is known about their biology due to difficulty accessing their natural habitat and obtaining live specimens. Although they are a frequent bycatch product in commercial bottom trawl fisheries, individuals of this species have rarely been raised in captivity and their embryonic development has not yet been documented. Considering these limitations, we were fortunate to have witnessed this process leading to successful hatching in an aquarium setting. Here, we present a brief observational account of the first‐known record of brooding, development, and hatching of fertilized eggs for *O. californicus*. The incubation time was a maximum of 10 months at a temperature between 8–10°C and embryos hatched over a period of 2.5 months. While more detailed research is needed, this preliminary information contributes to our limited knowledge of this species and supports life history theories of prolonged embryonic development under colder temperatures.

## Graphical abstract

The North Pacific bigeye octopus, *Octopus californicus* (Berry, 1911) is a cold‐water, deep‐sea octopod. Little is known about their biology due to difficulty accessing their natural habitat and obtaining live specimens. Here, we present a brief observational account of the first‐known record of brooding, development, and hatching of fertilized eggs for *O. californicus*.

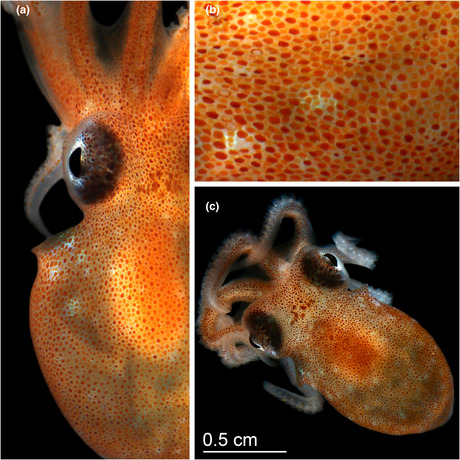

*Octopus californicus* (originally named *Polypus californicus* and commonly known as the “bigeye octopus”), was first described by S. Stillman Berry in 1911 based on a sample obtained off San Diego, California via dredging by the U.S.S. *Albatross*. Berry (1912) noted its short and stout body, partially‐webbed arms, and moderately‐sized eyes. Over the next century, there were sparse mentions of *O. californicus* in the scientific literature, although mitochondrial DNA genome sequencing identified it as a close relative to *Enteroctopus* spp. (Barriga‐Sosa et al., 1995; Ibáñez et al., 2014; Strugnell et al., 2011). Pacific fisheries have reported *O. californicus* collected via bottom trawls between 100 to 1000 m depth off California (Smith & Gordon, 1948), British Columbia (Gillespie et al., 1998), and the Gulf of Alaska (Ormseth & Conners, 2017). It has been suggested that *O. californicus* is the most common deep‐water octopod off the coast of California and has important market potential in commercial bottom trawl fisheries as a bycatch product (Hochberg, 1997), yet few observations exist about its life history.

In April 2021, an adult female bigeye octopus was haphazardly caught in a line trap set between 200 to 250 m depth offshore San Diego, California. She was transported to the experimental aquarium facilities at Scripps Institution of Oceanography, UC San Diego for the purposes of education and outreach. The octopus was held in a large open tank with chilled flow‐through seawater (8–10°C) aerated via an air stone and fed thawed fish, crustaceans, or mollusks. Beginning on 28 May 2021, the octopus laid eggs against one corner of the tank, just below the water surface, over the course of a few days. There were 200 eggs total, laid singly but arranged in multiple clumps. They were oblong, each about 2.02 ± 0.20 cm long and 0.79 ± 0.08 cm wide at the base, with a chorion stalk length of 0.52 ± 0.07 cm (mean ± SD; Table 1). They had a translucent milky color with green adhesive mucus at the tip of the stalk (Figure 1a). The female octopus continued eating while tending to the eggs; she rotated between them, cleaning them with her suckers throughout the day and blowing water over them with her funnel. Eggs were thought to be unfertilized since, as the only octopus in the tank, she could not have mated in captivity. However, exactly 4 months later on 28 September 2021, eyespots were visible in each egg (Figure 1b). The octopus must have mated in the wild and stored the sperm, which remained viable for at least 1 month. Sperm‐storing has been observed in many cephalopods; for example, female *O. vulgaris* have been known to store viable sperm for up to 10 months (Mangold, 1987).

**TABLE 1:** Morphological measurements of *Octopus californicus* eggs

	Mean ± SD (cm)	Range (cm)	*N*
Egg length	2.02 ± 0.20	1.77–2.48	24
Egg width	0.79 ± 0.08	0.65–0.95	24
Chorion stalk length	0.52 ± 0.07	0.42–0.65	24

**FIGURE 1:**
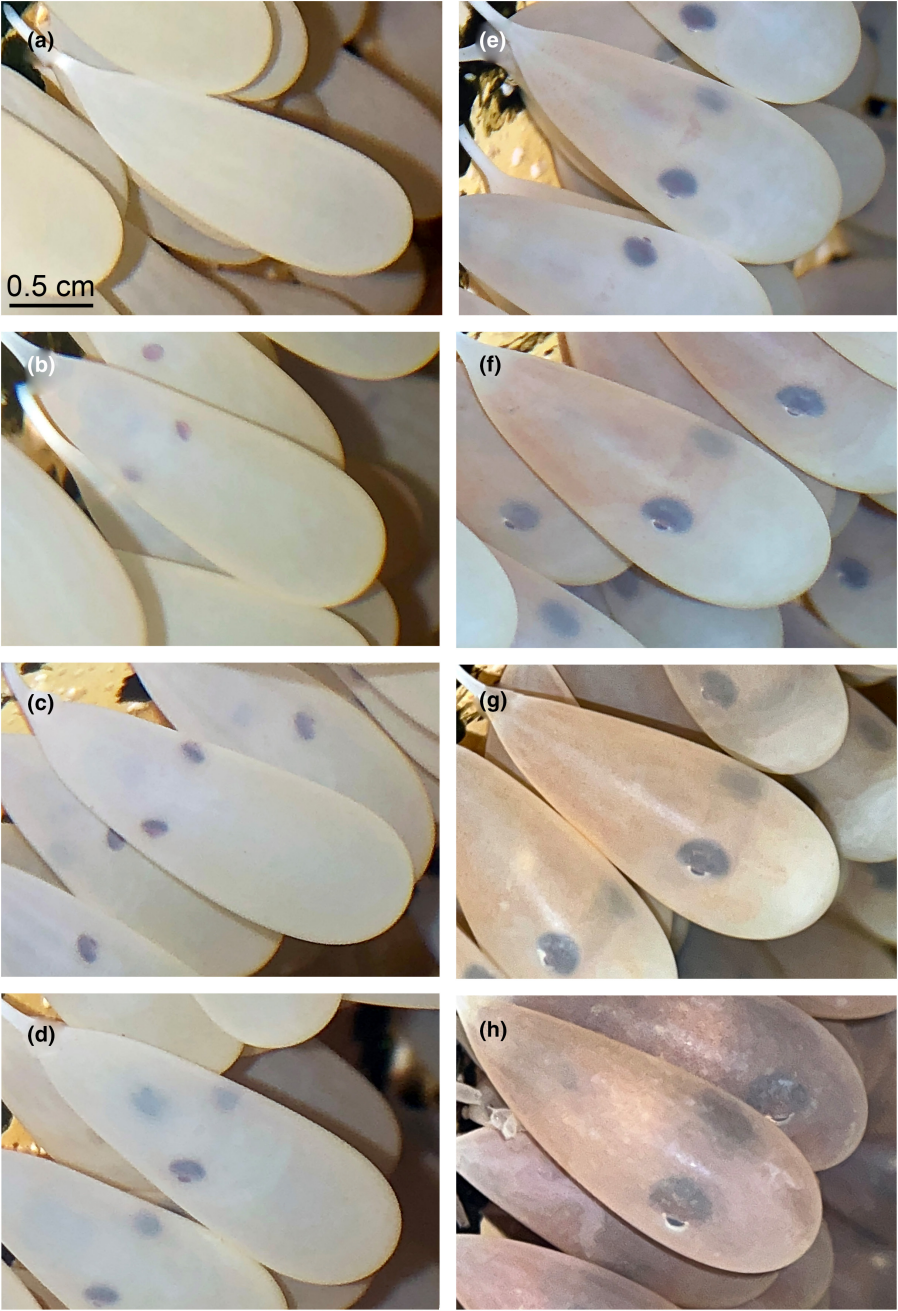
*Octopus californicus* development, showing eggs (a) initially, (b) at 4 months with eyespots, (c) at 4.5 months, (d) at 5 months with unidentified organs below the eyes, (e) at 5.5 months; note the patch of chromatophores between the eyes, (f) at 6 months with more pigmentation in the mantle, (g) at 7 months; note remaining yolk, and (h) at 8 months, just prior to hatching. Scale bar represents 0.5 cm. Photos by A. Khen.

Week‐by‐week, the eyespots in the eggs became larger and more three‐dimensional, with reflective irises (Figure 1c). By late October 2021, an unidentified organ began to develop as a dark spot below the eyes (Figure 1d). On 28 October 2021, tragically and unexpectedly, the mother octopus crawled out of the tank overnight and died. We had been keeping individuals of this species in open tanks for years but have only seen two escape attempts, both by brooding females (the former with unfertilized eggs). While we cannot be certain of the cause of death, this incident may have been triggered by the optic gland or other hormones which control octopus sexual reproduction and senescence (Wang et al., 2022). In classic experiments where the optic gland was surgically removed, brooding female octopuses have been shown to resume feeding, abandon their eggs, and live for longer (Wodinsky, 1977), otherwise they typically die when the eggs hatch. In captivity, there have been records of starvation, erratic behavior, and physical deterioration preceding maternal death (Anderson et al., 2002; Wang et al., 2022), however we did not notice any signs of decline; the mother octopus had been eating, behaving, and interacting with her caretakers normally. We were concerned that the mother's death would have a negative effect on the embryos, but a recent study found that *O. vulgaris* embryos could successfully develop without maternal care in artificial oxygenated seawater at a continuous strong flow rate (Deryckere et al., 2020). A webcam was set up to monitor the eggs remotely and aeration was increased to simulate movement by the mother's arms and prevent debris, bacterial, or fungal overgrowth. DNA sequencing of the mitochondrial Cytochrome oxidase subunit I (COI) from a tissue sample of the deceased octopus confirmed a strong match (BLAST 99.7%) with other COI sequences for *O. californicus* available on GenBank (GenBank accession # OM753096; https://www.ncbi.nlm.nih.gov/nuccore/OM753096). The octopus was preserved in ethanol and added to the Benthic Invertebrate Collection at Scripps (catalog #SIO‐BIC M18619).

The embryos continued to develop in their mother's absence with constant water flow, aeration, and monitoring. Chromatophores first appeared in small patches between the eyes and populated the rest of the body, including the mantle and arms, by around 5.5 months (Figure 1e,f). Mantles and arms were brownish‐orange (Figure 1g), yolk was white, and at this point some eggs contained less yolk than others; presumably, not all embryos were absorbing yolk at the same rate. As embryos were approaching their final developmental stages, slight movements could be seen from within the eggs (Video S1). Although some of the embryos had inked, the ink would diffuse through the outer membrane of the egg cases and this did not seem to inhibit further development. On the night of 7 January 2022, after more than 7 months of incubation, the first few hatchlings were born (Video S2).

The early hatchers still had some yolk sac remaining and this was consumed or lost within the first day. Hatchlings had a mantle length of 1.09 ± 0.06 cm, mantle width of 0.78 ± 0.05 cm, head width of 0.73 ± 0.04 cm, and 0.33 ± 0.02 cm eye diameter (Table 2). Their arms were up to ~1 cm long with approximately 60 suckers per arm in rows of two, with webbing halfway down their arms. They were able to camouflage from pale to dark orange and would ink when startled, raising their papillae. They had numerous small reddish‐brown chromatophores (at a density of >50 chromatophores per mm^2^) in no conspicuous arrangement, with 7–9 larger chromatophores between the eyes on the head (Figure 2). There was no planktonic juvenile phase and the hatchlings resembled and mostly functioned as miniature adults (Robin et al., 2014), although hatchlings inked more frequently than adults observed in captivity (pers. obs.). Hatchlings were transferred to a kreisel nursery tank covered in black vinyl, supplied with chilled, aerated flow‐through seawater (~8°C) and were fed live amphipods and mysid shrimp. We also placed plastic tubes at the bottom of the tank, where most of the hatchlings settled; while they could swim, they were predominantly benthic.

**TABLE 2:** Morphological measurements of *Octopus californicus* hatchlings

	Mean ± SD (cm)	Range (cm)	*N*
Mantle length	1.09 ± 0.06	0.99–1.20	20
Mantle width	0.78 ± 0.05	0.71–0.86	20
Head width	0.73 ± 0.04	0.67–0.82	20
Eye diameter	0.33 ± 0.02	0.29–0.38	20

**FIGURE 2:**
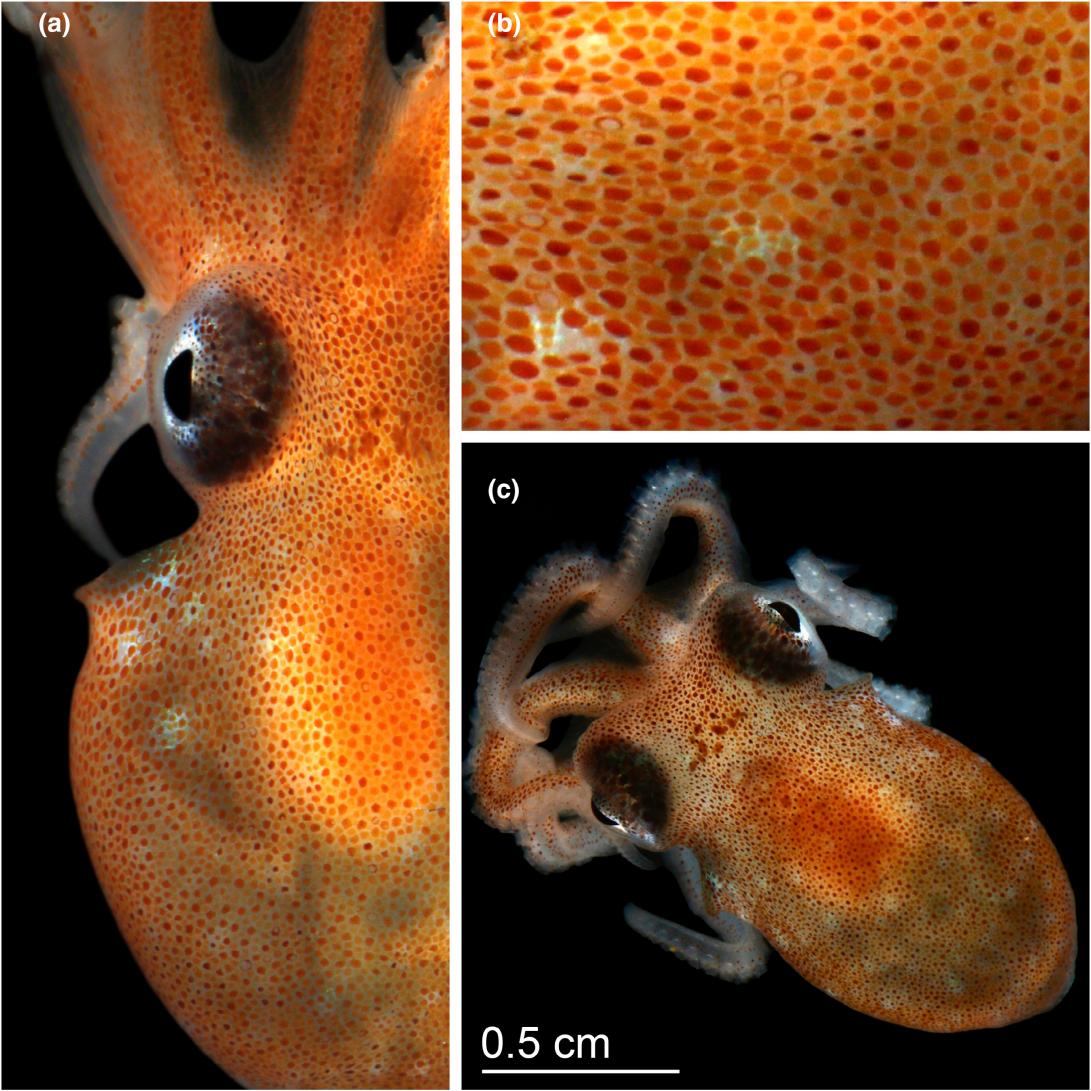
(a) Lateral view, (b) chromatophore closeup, and (c) top view of a 1‐week‐old *Octopus californicus* hatchling. Scale bar represents 0.5 cm. Photos by G.W. Rouse.

A month after the initial hatch date, there were only around 10 hatchlings total with the majority of eggs still unhatched. Some of the early hatchers survived but most had died within weeks, possibly from prematurity. Later, we suspected the early hatchers were not strong enough to catch live prey since we had not witnessed any of them eating. We started offering them freshly‐killed amphipods and they had greater survival. At 8 months incubation, there was no visible yolk left in the rest of the eggs (Figure 1h). By 23 March 2022, all individuals had hatched, with up to 10 new hatchlings per day in the final weeks. The delay in subsequent hatching is consistent with the flexible hatching timeline proposed by Boletzky (2003), which is based on “hatching competence” (rather than a specific phase where hatching occurs) and is thought to maximize post‐hatch survival. There did not appear to be a correlation between the eggs that were laid first and those that hatched first, since all eggs were laid within a matter of days, yet hatching was spread out over more than 2.5 months. The mid‐ and late hatchers could hunt on their own and were fed frozen krill or fish at least once a day. Groups of up to 20 juveniles were kept in the same tank with no evidence of cannibalism and near‐complete survival. While the lifespan of this cold‐water species is as of yet unknown, at 6 months old their mantle length was only up to ~1.5 cm. Given that adults' mantle lengths can be 12–14 cm (Hochberg, 1997), perhaps they have a rather long lifespan compared with warm‐water species.

Overall, eggs of this species exhibited a remarkable incubation time of nearly 10 months maximum. This could be related to low temperatures, which slows their metabolism (Boletzky, 1994) and is known to prolong embryonic development (Villanueva & Norman, 2008). Another deep‐sea octopod, *Graneledone boreopacifica*, observed via a remotely‐operated vehicle in Monterey Submarine Canyon, California, where temperature was colder at about 3°C, had a 53‐month egg‐brooding period (Robison et al., 2014). Life history traits such as the large size of the eggs and relatively low fecundity were likely also factors in the extended development period (Ibáñez et al., 2021). Further, the staggered hatching may either be a strategy to maximize post‐hatch survival or a result of multiple paternity, prematurity, and/or triggered by disturbance. After the juveniles were well‐established at several months old, we distributed them to public aquaria across California, where they would serve as “animal ambassadors” educating the general public about this species. Ultimately, this will have been the first‐known opportunity to observe the brooding, development, and hatching of fertilized eggs for this common yet understudied deep‐water octopod.

## AUTHOR CONTRIBUTIONS

**Adi Khen:** Investigation (equal); writing – original draft (lead). **Lillian R. McCormick:** Resources (supporting); supervision (supporting); writing – review and editing (equal). **Christine A. Steinke:** Supervision (equal); writing – review and editing (supporting). **Greg W. Rouse:** Resources (supporting); writing – review and editing (equal). **Phil J. Zerofski:** Resources (lead); supervision (lead).

## Supporting information


Video S1

Video S2

## Data Availability

The mitochondrial cytochrome oxidase subunit I of the *Octopus californicus* studied here is available at the National Center for Biotechnology Information (https://www.ncbi.nlm.nih.gov/nucleotide/) under accession number OM753096 (https://www.ncbi.nlm.nih.gov/nuccore/OM753096). The voucher specimen of the octopus is held at the Scripps Institution of Oceanography Benthic Invertebrate Collection (https://sioapps.ucsd.edu/collections/bi/) under catalog number M18619.
